# Central Diabetes Insipidus Following Immunization With BNT162b2 mRNA COVID-19 Vaccine: A Case Report

**DOI:** 10.3389/fendo.2022.889074

**Published:** 2022-05-04

**Authors:** Bruno Bouça, Marisa Roldão, Paula Bogalho, Luís Cerqueira, José Silva-Nunes

**Affiliations:** ^1^ Department of Endocrinology, Diabetes and Metabolism, Centro Hospitalar Universitário de Lisboa Central, Lisbon, Portugal; ^2^ Department of Nephrology, Centro Hospitalar do Médio Tejo, Torres Novas, Portugal; ^3^ Department of Neuroradiology, Centro Hospitalar Universitário de Lisboa Central, Lisbon, Portugal; ^4^ Nova Medical School/Faculdade de Ciencias Medicas, Universidade Nova de Lisboa, Lisbon, Portugal; ^5^ Health and Technology Research Center (H&TRC), Escola Superior de Tecnologia da Saude de Lisboa, Lisbon, Portugal

**Keywords:** case report, diabetes insipidus, COVID-19, vaccine, mRNA

## Abstract

**Introduction:**

Cases of central diabetes insipidus (CDI) have been reported after COVID-19 infection, with hypophysitis being the most likely cause. COVID-19 vaccines potential adverse effects may mimetize some of these complications.

**Case Report:**

Woman 37 years old, with rheumatoid arthritis under adalimumab (40 mg twice a month) since December 2018. She was in her usual state of health when she has received the second dose of BNT162b2 mRNA COVID-19 vaccine (June 2021). Seven days later, she started reporting intense thirst and polyuria and consulted her family physician.

**Blood Analysis:**

creatinine 0.7 mg/dL, glucose 95mg/dL, Na+ 141mEq/L, K+ 3.9 mEq/L, TSH 3.8 mcUI/L (0.38-5.33), FT4 0.9 ng/dL (0.6-1.1), cortisol 215.4 nmol/L (185-624), ACTH 21.9 pg/mL (6- 48), FSH 4.76 UI/L, LH5.62 UI/L, estradiol 323 pmol/L, IGF1 74.8 ng/mL (88-209), PRL 24.7mcg/L (3.3-26.7) osmolality 298.2 mOs/Kg (250- 325); Urine analysis: volume 10200 mL/24h, osmolality 75 mOs/Kg (300-900), density 1.002. On water restriction test: 0’ – Serum osmolality 308.8mOsm/Kg vs. urine osmolality 61.0 mOsm/Kg; 60’ - urine osmolality 102 mOsm/Kg; urine osmolality 1 h after desmopressine was 511mOsm/kg. MRI revealed no abnormal signs consistent with hypophysitis except for the loss of the posterior pituitary bright spot on T1 weighted imaging. Diagnosis of CDI was assumed, and started therapy with desmopressine. A report of potential adverse effect was addressed to national health authorities.

**Conclusion:**

In hypophysitis MRI often shows loss of posterior pituitary bright spot on T1 weighted imaging, pituitary enlargement or stalk thickening but those findings were not present in this patient. To the best of our knowledge, CDI has never been reported following administration of a COVID-19 vaccine.

## Introduction

SARS-CoV-2 infection (COVID-19) is asymptomatic in a large number of individuals. In symptomatic patients, infection mainly causes lung disease and can be complicated by acute respiratory distress syndrome. Some other documented complications include myocarditis, pericarditis and thromboembolic events. However, complications involving the endocrine system have been increasingly described, which can be explained by the presence of ACE2 receptors in endocrine glands (1).

As a result of the rapid spread of this virus, there was an urgent need to develop and approve vaccines that would reduce COVID-19-associated morbidity and mortality. Although the notion of mRNA vaccines has been scientifically relevant since the early twenty-first century, the development of the COVID-19 vaccines marks the first large-scale deployment of this sort of vaccination (2). mRNA vaccines offer a new and different approach to pathogen immunity, delivering the relevant antigen’s genetic code. This mRNA is subsequently translated by the host to produce the protein of the pathogen (3, 4). The most frequently reported adverse events associated with those mRNA vaccines include myalgia, arthralgia, nausea, tiredness, and dermal reactions at the injection site (5). Due to its rapid and global use, other rare adverse effects have been reported.

Central Diabetes Insipidus (CDI) is a rare condition in which the neurons of the hypothalamus/posterior pituitary axis are destroyed, resulting in a decrease of arginine vasopressine (AVP) synthesis/secretion in response to osmotic stimulation, affecting both men and women at any age, leading to a large urine output. A wide range of acquired or congenital lesions can produce the condition, and it is often caused by traumatic injury to the hypothalamus or posterior pituitary gland, as well as destruction/degeneration of neurons originating in the supraoptic and paraventricular nuclei of the hypothalamus, as a result of surgery or head injury. This disease has been found in a number of conditions, including inflammatory or traumatic local damage, as well as in the idiopathic form. Usually, symptoms such as polydipsia, polyuria and nocturia develop when more than eighty percent of AVP-secreting neurons are damaged, with the severity of resultant hypotonic diuresis being dependent on the extent of the neurohypophyseal damage (6, 7). When hypotonic polyuria is diagnosed, differential diagnosis between CDI, nephrogenic DI, and primary polydipsia, is critical. Water deprivation test is usually used for determining the cause of polyuria–polydipsia syndrome. This test is based on an indirect assessment of AVP activity using urine concentration measurements during a lengthy period of dehydration and again following injection of desmopressin, an exogenous synthetic AVP analogue. The test results are then interpreted according to published recommendations. Because the purpose of therapy is to improve symptomatology, the suggested regimen should be adjusted to each patient’s specific needs. The therapy regimen’s safety and the avoidance of harmful side effects from overtreatment are the most important factors, as CDI usually has a mild course. Because of its lengthy half-life, selectivity for AVPR2, and availability of numerous preparations, desmopressin is the current standard of therapy for patients with CDI. Patients frequently choose oral formulations since they are more convenient (7).

We present the report of a woman, who developed CDI one week after the second dose of BNT162b2 mRNA COVID-19 vaccine (BioNTech/Pfizer), with evidence of infundibuloneurohypophysitis on MRI.

## Case Description

A 37-year-old female patient, with previous diagnosis of rheumatoid arthritis, was under well tolerated therapy with adalimumab (40 mg twice a month since December 2018). She had no other relevant medical, surgical or familial history. She was in her usual state of health when she has received the second dose of BNT162b2 mRNA COVID-19 vaccine (June 2021). Seven days later, she started reporting intense thirst and polyuria and consulted her family physician. She denied headache, polyphagia, weight loss, foamy urine, macroscopic hematuria, peripheral or periorbital edema. On physical examination: blood pressure 120/80 mmHg, heart rate 70 bpm, weight 60 kg and height 165 cm; cardiac and pulmonary auscultation were normal and she had no relevant abnormalities. She was then referred to the Nephrology Department of her local hospital.

Blood analysis: creatinine 0.7 mg/dL, glucose 95 mg/dL, Na^+^ 141mEq/L, K^+^ 3.9 mEq/L, Calcium 8.9 mg/dL, Albumin 42 g/dL, TSH 3.8 mcUI/L (0.38-5.33), FT4 0.9 ng/dL (0.6-1.1), cortisol (8 am) 215.4 nmol/L (185-624), ACTH 21.9 pg/mL (6-48), osmolality 298.2 mOs/Kg (250-325); Urine analysis: volume 10200 mL/24h, osmolality 75 mOs/Kg (300-900), density 1.002.

Due to these findings, diabetes insipidus was suspected and she was then admitted to the Nephrology ward to perform a water deprivation test. This test is based on an indirect assessment of AVP activity using urine concentration capacity measurements during a lengthy period of dehydration and again after injection of desmopressin. Hourly measurements of body weight and urine osmolality are taken during water restriction until 2–3 samples differ by less than 30 mOsm/kg, or the patient loses more than 3% of his or her body weight, or plasma Na+ surpasses 145 mEq/L. Desmopressin is then injected. The osmolality of the urine is tested 60 minutes later. CDI and nephrogenic DI are distinguished by their responses to desmopressin treatment. Complete nephrogenic DI is diagnosed when urine osmolality remains below 300 mOsm/kg after thirst and does not increase by more than 50% following desmopressin administration. If the urine osmolality rises by more than 50% following desmopressin administration, complete CDI is identified. Urinary concentration rises to 300–800 mOsm/kg in partial CDI and primary polydipsia, with increments of >9% (in partial CDI) and 9% (in primary polydipsia) after desmopressin injection (8).

**Figure 1:**
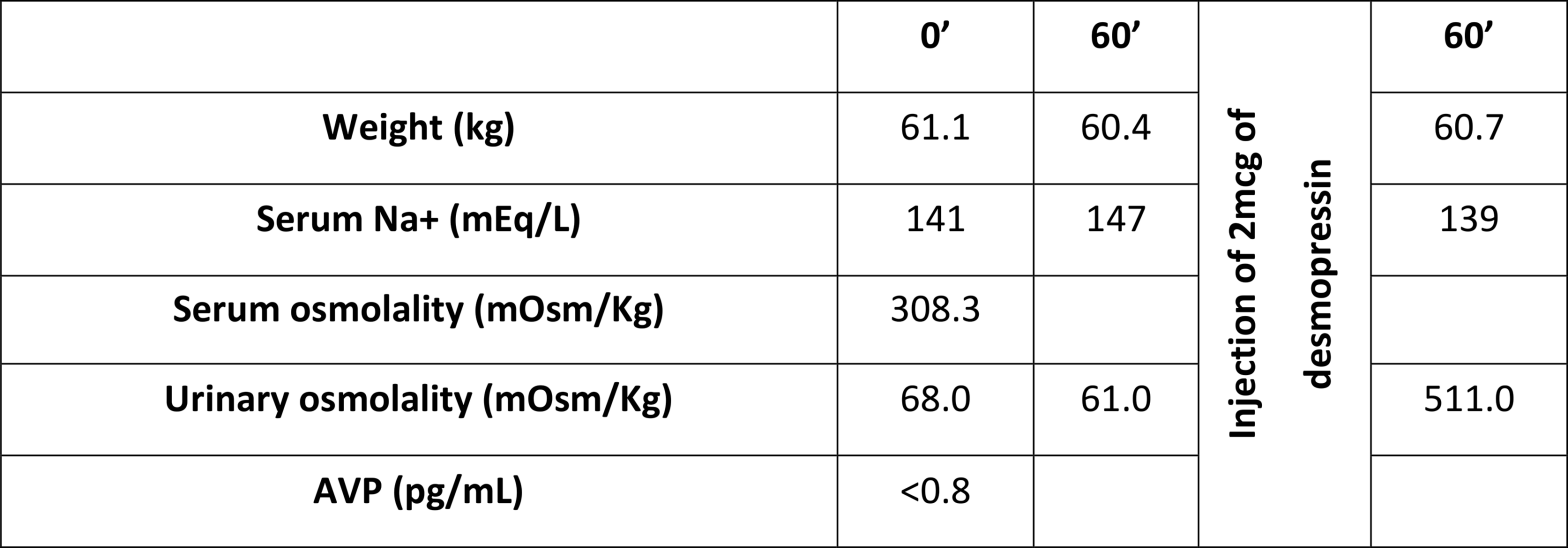
Water deprivation test results.

**Figure 2:**
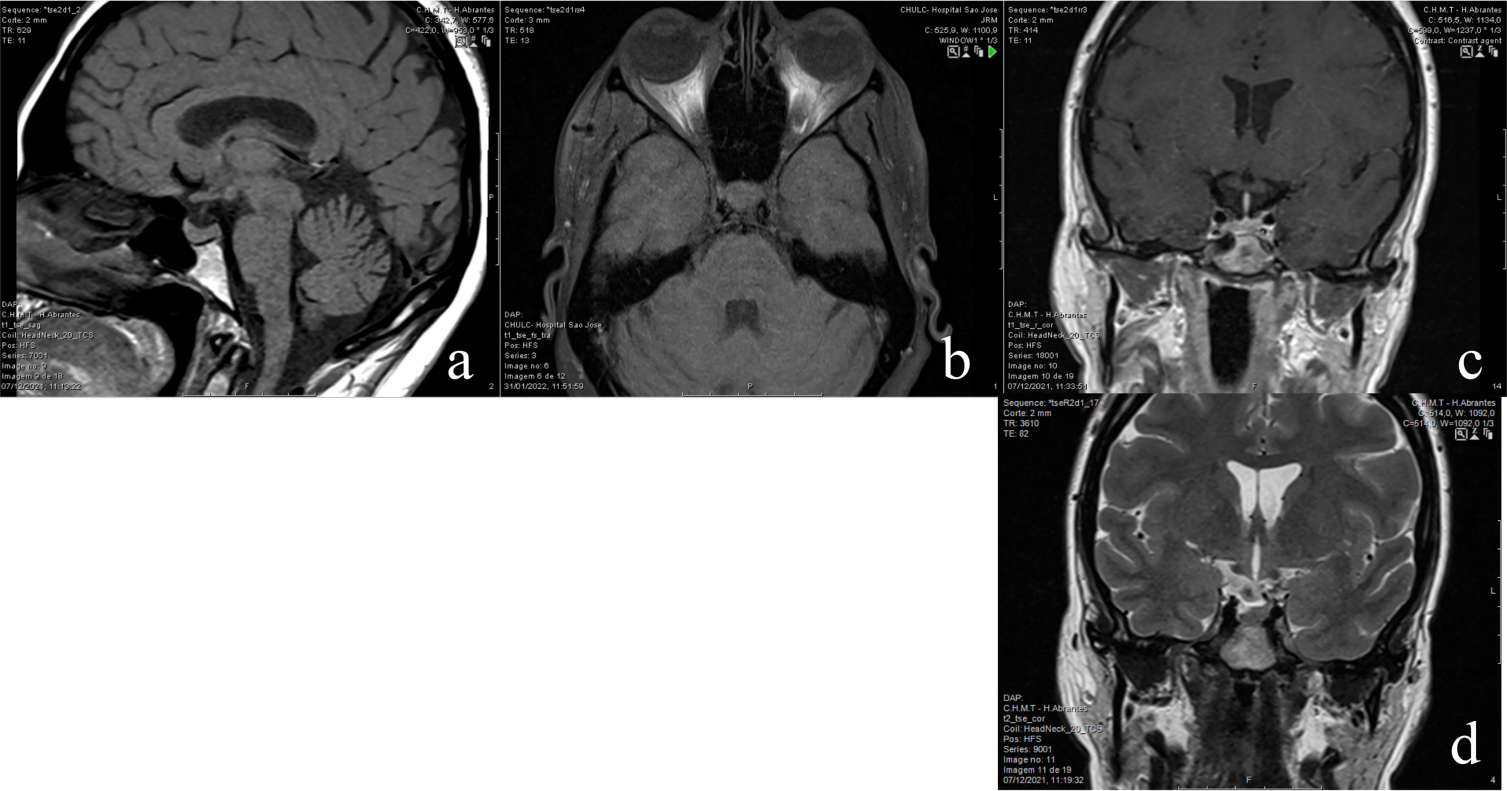
**(A)** sagital T1 showing a normal anterior pituitary gland and absent posterior pituitary hyperintensity; **(B)** axial T1 fat-sat confirms absent posterior pituitary hyperintensity; **(C)** - coronal T1 post gadolinium depicts the normal shape and height of the enhancing anterior pituitary gland and pituitary stem; **(D)** - coronal T2 depicts the normal hypothalamic structures.

The water deprivation test started at 08.00 a.m. of the day after admission. The results showed: serum basal Na+ 141 mEq/L; serum basal osmolality of 308.8 mOsm/Kg; serum basal AVP below detection limit of 0.8 pg/mL; basal urine osmolality: 68.0 mOsm/Kg; At 60 min. Na+ 147 mEq/L and urine osmolality 61 mOsm/Kg; 1 h after 2 mcg of intravenous desmopressin: urine osmolality was 511 mOsm/Kg and Na+ 139 mEq/L. (**Figure 1**) The Endocrinology Department was contacted for clinical guidance. Further hormonal analysis showed: FSH 4.76 UI/L, LH 5.62 UI/L, estradiol 323 pmol/L, IGF1 74.8 ng/mL (88-209), PRL 24.7 mcg/L (3.3-26.7). MRI of the pituitary gland revealed loss of the posterior pituitary bright spot on T1 weighted imaging. (**Figure 2**) Diagnosis of CDI was assumed, and she started therapy with oral desmopressin 0.06 mg twice a day. Although pituitary biopsy was not conducted, other probable causes of CDI were ruled out – serum levels of iron, IgG4, angiotensin-converting enzyme and beta2-microglobulin were normal. Infection by mycobacterium tuberculosis was also ruled out. A report of this potential adverse effect from BNT162b2 mRNA COVID-19 vaccine was addressed to national health authorities. On the last appointment (December 2021), she was under oral desmopressin 0.06 mg three times a day, had no polydipsia or polyuria, blood pressure was 110/80 mmHg, and analytical results showed a serum osmolality of 297.2 mOsm/kg, and urine osmolality of 148.0 mOsm/kg. Desmopressin was then titrated to 0.12 mg twice a day. Reevaluation of anterior pituitary function was normal: TSH 2.62 mcUI/L, FT4 0.89 ng/dL, cortisol 8 a.m. 302 nmol/L, IGF1 78 ng/mL, FSH 5.7 UI/L, LH 5.8 UI/L, estradiol 412 pmol/L.

## Discussion

The possibility of a direct causal relationship between vaccine administration and the development of diabetes insipidus was raised, not only by the suspicious temporal relationship and the exclusion of other possible causes of hypophysitis, but also in the absence of any other symptoms, such as headache or visual change. Furthermore, although being one of the most common causes (72%), the existence of isolated CDI and the maintenance of adenohypophysis function make the diagnosis of lymphocytic hypophysitis exceedingly doubtful (9).

Although another example of hypophysitis with hypopituitarism – central hypocortisolism and central hypothyroidism - has already been recorded following immunization with the mRNA-1273 COVID-19 vaccine, this is the first report of CDI reported after immunization with the BNT162b2 mRNA COVID-19 vaccine (10).

In the literature, three cases of diabetes insipidus have been reported 4 to 5 weeks after COVID-19 diagnosis. In hypophysitis, MRI often shows, along with loss of posterior pituitary bright spot on T1 weighted imaging, pituitary enlargement or stalk thickening but those findings were not present in this patient. However, the same lack of pathological signs of hypophysitis in MRI has also been reported in 2 of these cases, but in the other it was possible to verify suspicious signs of infundibuloneurohypophysitis (11–13). Authors of these clinical reports believed that direct hypothalamus injury or inflammatory hypophysitis were the most likely explanations for this pathophysiological feature. Furthermore, it is known that ACE-2 receptors are expressed in both the pituitary and the hypothalamus, and that these receptors play a role in COVID-19 pathogenesis (1). Hypothalamic neuronal degeneration has also been found in autopsied COVID-19 individuals. Along with other adverse effects occurring after administration of mRNA vaccines, delayed reactions have been reported in skin biopsies; it was shown infiltration of CD4 + and CD8 + lymphocytes and eosinophils which may imply a more broad immune reaction (14, 15).

In conclusion, we believe that CDI, as reported in cases of SARS-CoV2 infection, can also be a serious but rare side effect of mRNA vaccination. To the best of our knowledge, CDI has never been reported following administration of a COVID-19 vaccine.

## Data Availability Statement

The original contributions presented in the study are included in the article/supplementary material. Further inquiries can be directed to the corresponding author.

## Ethics Statement

Written informed consent was obtained from the individual(s) for the publication of any potentially identifiable images or data included in this article.

## Author Contributions

BB - follow-up of the patient and writing manuscript MR - follow-up of patient and writing manuscript; PB - follow-up of patient and revising manuscript; LC - neurorradiologist responsible for organizing images and revising manuscript; JS-N - revised the manuscript. All authors contributed to the article and approved the submitted version.

## Conflict of Interest

The authors declare that the research was conducted in the absence of any commercial or financial relationships that could be construed as a potential conflict of interest.

## Publisher’s Note

All claims expressed in this article are solely those of the authors and do not necessarily represent those of their affiliated organizations, or those of the publisher, the editors and the reviewers. Any product that may be evaluated in this article, or claim that may be made by its manufacturer, is not guaranteed or endorsed by the publisher.
